# Impact of corrupt admission on the mental health of Chinese adolescents

**DOI:** 10.1038/s41598-024-67096-5

**Published:** 2024-07-15

**Authors:** Hongbin Yuan, Danyang Li, Feiran Yang, Zhijian Zhang

**Affiliations:** 1 School of Economics, Zhejiang Gongshang University, Xuezheng Street, Hangzhou, 310018 Zhejiang China; 2 Xukou Middle School, Qingming Road, Suzhou, 215164 Jiangsu China; 3 School of Marx, Jianghan University, Sanjiaohu Street, Wuhan, 430056 Hubei China

**Keywords:** Corruption, Adolescent, Mental health, Junior high school, Admission, Health care economics, Risk factors

## Abstract

Through preferential treatment by education officials or through bribery, some adolescents can obtain admission to a junior high school. However, it is unclear whether it affects the mental health of adolescents. This study used Propensity Score Matching to examine the effects of corruption on adolescent mental health. A total of 17,254 junior high school students sample (11–18 years old; 48.7% girls and 53.1% boys) were used from the China Education Panel Survey. 14.1% of adolescents attended a junior high school by corrupt means, corruption had a significantly negative effect on the mental health of these adolescents (ATT = −0.388, *p* < 0.01), the reasons grounded in the fact that they received more criticisms from teachers and wanted to leave their current school. In general, corruption in the admissions process can have detrimental effects on the mental health of adolescents. This study extends the previous articles on how to improve adolescent mental health and complements the application of cognitive dissonance theory. Findings from this study revealed that anti-corruption in the education sector is necessary, and the institutional design to ensure fair enrolment in basic education will contribute to the mental health of adolescents.

## Introduction

The phenomenon of corruption is a salient issue across multiple domains in developing nations and gaining increasingly studied due to its huge impacts on economic growth and individual development^1–6^. Corruption in the education sector is a question worth exploring, as it is closely related to the human capital accumulation of school-age adolescents. In China, due to the extreme scarcity of high-quality education resources and institutional imperfections, corruption is frequently observed in the school admission process. Through preferential treatment by education officials or through bribery, some parents can secure admission for their children into schools for which they would not otherwise meet the eligibility criteria. This kind of corruption is common in Chinese education sectors and may potentially impact the mental health of adolescents. As indicated by the Cognitive Dissonance Theory, when there is inconsistency between an individual's beliefs and actions, psychological discomfort may arise^7^. However, there have been few studies examining its possible effects on adolescent mental health. This research aims to fill this gap by exploring the effect of corruption in the admission process of junior high schools on the mental health of adolescents.

Previous literature has established a link between corruption and mental health. In Vietnam, it has been found that corruption causes a significant decline in revenue and thus has a negative impact on residents' mental health^2^. Furthermore, studies conducted in Europe and Africa have suggested a negative association between corruption and mental well-being^8,9^. While these studies are useful in understanding the impact of corruption on adult mental health, the existing literature on education corruption mainly focused on its correlation with test scores or discusses anti-corruption policy^10–12^, and less is known about the effects of corruption in the school admission process.

In China, junior high school education constitutes the last three years of the compulsory nine years of education, and admission is primarily based on "*Xuequ*", which refers to the geographic distance between a student's family property and a specific school. If a family owns property within a certain distance of the school, their adolescent children become eligible for admission. *Xuequ* is designated by local governments, meaning that a family's geographic location ultimately determines their child's admission to junior high school. However, government leaders in charge of education affairs and school leaders have the power to expand the number of admissions, creating opportunities for corruption. Those who have close relationships with these leaders may use these connections or resort to bribery to enroll their children in desired schools.

Corruption may hurt adolescent mental health. Corruption implies a violation of social norms, which in turn incurs psychological costs^13^, negatively impacting the mental health of adolescents. According to cognitive dissonance theory, when individuals face conflicting beliefs and behaviors, they experience psychological discomfort or tension^7^. Adolescents who attend school through corrupt means may become aware of corruption as wrongdoing, it can inevitably lead to conflicting beliefs and behaviors that negatively impact their mental health. Additionally, students who gain admission through corrupt means are often perceived as having obtained unfair advantages. As a result, this may affect their relationships with classmates. Teachers, in their role as upholders of fairness, are more likely to criticize these students further in order to obtain psychological compensation. However, empirical research on this topic remains limited.

Based on a nationally representative survey sample, the current study utilized Propensity Score Matching (PSM) methods to empirically examine the effect of corruption in Chinese junior high school admissions on adolescent mental health and further explored the mechanisms behind it. To our knowledge, this is the first study that builds a correlation between corruption in junior high school admission and adolescent mental health.

## Methods

### Data

The data used in this study were drawn from 2013 to 2014 school year of the China Education Panel Survey (CEPS), a nationally representative junior high school survey conducted by Renmin University of China that aimed to investigate how adolescents’ development is affected by parents, school, and community. The survey was collected by a four-stage probability sampling design, including students of 7th and 9th graders, 19,487 students were randomly selected from 438 classes across 112 junior high schools in 28 counties of China.

In the CEPS, four types of questionnaires were used to collect information on students, their parents, teachers (including head teacher and main subject teachers like English, Chinese, and Math), and school administrators. We merged these four types of data and deleted the samples with missing values, totaling 2233, which accounted for 11.46% of the original sample size. Ultimately, the sample used in this study was 17,254 students from 7 and 9th grade.

### Measures

#### Mental health

The dependent variable is mental health, we referred to the measurement methods used in existing literature ^14,15^, which was measured by a series of questions related to adolescent psychological conditions. Specifically, in the CEPS, respondents were asked whether they had ever felt unhappy, depressed, anxious, sad, or had little interest in doing things in the past seven days. All of the responses were rated with five options included never (1), seldom (2), sometimes (3), most of the time (4), and always (5). In this study, we assigned high scores for enhanced adolescent mental health by reversing the values of five options and then summing up the five items to create a continuous variable. We also tested for internal consistency, with Cronbach's alpha value at 0.8551, indicating excellent consistency.

#### Corruption

The independent variable in this study refers to the corruption during the junior high school admission process, which implies a violation of the admission rules. In the CEPS, parents of adolescents were asked whether they had done any of the following to get their child into junior high school. Two options were related to corruption: “got a friend to help” and “bribed relevant officials”. The other options were not related to corruption and included: “pay extra” (Some local governments stipulate that migrant populations can obtain admission to certain schools by paying additional fees), “have children take various academic or specialty examinations”, “buy a house in the specific area where the school is located”, “change the household registration (*Hukou*) status”, “register the *Hukou* at a relative or friend’s residence”, and “none of above”. These options were considered permissible under the admission policy. A dummy variable was thus constructed to capture corruption, where the variable takes a value of 1 if either of the option “got a friend to help” or “bribed relevant officials” were selected, and a value of 0 if any other non-corrupt options were selected.

#### Covariates

In this study, we employed a series of covariates that might affect the relationship between adolescent mental health and its determinants. These control variables encompassed individual-level covariates such as age, gender, nationality, test scores, and grade level. At the family level, we included covariates related to economic conditions and parents' education level. Furthermore, we also considered class and school-level covariates, including the number of students in a class, class rank within a school, and school rank within the district.

#### Mechanism variables

Four mechanism variables were used in this study: interpersonal relationships, students receiving criticism from teachers, parents receiving criticism from teachers, and students' desire to leave school. For interpersonal relationships, the CEPS student questionnaire included the question “How many best friends do you have”. Students were asked to answer the number of best friends they have. We used this quantity to represent students' interpersonal relationships, as having more friends implies better interpersonal relationships for students. Regarding students receiving criticism from teachers and parents receiving criticism from teachers, the CEPS student questionnaire includes the following two questions: “In terms of school life, do you agree with the following statement: My head teacher often criticizes me”, and “Regarding school life, do you agree with the following statement: My parents often receive criticism from teachers about me”. The response options for both questions are: strongly disagree (1), disagree (2), agree (3), strongly agree (4). We use the responses to this question to measure teacher criticism, higher scores indicate that students or parents perceive more teacher criticism. Moreover, for students' willingness to leave the school, in the CEPS student questionnaire, there is a question: “Regarding school life, do you agree with the following statement: I wish to attend another school”. The response options include: completely disagree (1), somewhat disagree (2), somewhat agree (3), completely agree (4). We used the responses to this question to measure students' willingness to leave the school, with higher values indicating a greater desire among students to leave their current school.

### Empirical strategy

Comparing the mental health of adolescents admitted to a school through corrupt means to those admitted through non-corrupt means would not allow for an accurate assessment of the effect of corruption on mental health. Factors that determine mental health may indeed be correlated with the choice of corruption, leading to selection into the treatment. In this situation, standard regression methods are not recommended, as it assumes that covariates follow a common distribution and functional form across treatment and control groups^16^. Therefore, we exploited Propensity Score Matching (PSM) to estimate the effect of corrupt admission on adolescent mental health, which is a suitable method to obtain robust causal effects in this situation for selecting comparable treatment and control samples from given characteristics^17^.

PSM relies on the assumption that the treatment is exogenous, the differences between the treatment group and the control group are due to the treatment^17^. In this study, the treatment means those who attend a junior high school through corruption. It is worth noting that we use non-experimental data, and the treatment is not totally exogenous. For example, those with a wealthy family background are more likely to benefit from corrupt admission, and the economic status of the family has been proven to be correlated with adolescents’ mental health^18,19^. The advantages of PSM lie in its conditional independence assumption (CIA), which is helpful to tackle the aforementioned challenge. To make the CIA more plausible, propensity scores are first generated to enable subjects with similar scores to be comparable. The propensity score represents “the conditional probability of assignment to a particular treatment given a vector of observed covariates”^17^. Therefore, the first step of PSM is to estimate the propensity score using logit regression based on a series of observed covariates.

The second step of PSM is to choose a matching algorithm, which means how to match treatment individuals (those attending a junior high school through corrupt means) and control individuals, for which the treatment effects are therefore been estimated. In this study, we use four kinds of matching algorithms. The first one is nearest neighbour (NN) matching, the individual from the control group is chosen as a matching partner for a treated individual that is closest in terms of the propensity score. We use 1:1 nearest neighbour matching and 1:4 nearest neighbour matching respectively, that is, the nearest one and the nearest four from the control group are selected to match a treatment individual. Nearest neighbour matching is not perfect, it faces the risk of bad matches if the closest neighbour is far away^20^. Therefore, to ensure the robustness of our estimates, we exploited more matching algorithms including caliper matching and kernel matching. Applying caliper matching means that an individual from the comparison group is chosen as a matching partner for a treated individual that lies within the caliper (propensity range)^20^. A variant of caliper matching named radius matching is recommended^21^. The basic idea of this variant is to use not only the nearest neighbour within each caliper but all of the comparison members within the caliper, bad matches are avoided due to many comparison units within the caliper are available for matching. The caliper width is 0.1 in this study, implying the samples within 0.1 in terms of the distance of propensity score from the control group are selected to match a treatment sample. Kernel matching can be seen as a weighted regression of the counterfactual outcome on an intercept with weights given by the kernel weights^22^.

Once the after-match control group was constructed, we could calculate the average treatment effect for the treated (ATT). In this study, it means the difference between the average mental health for adolescents who attend junior high school through corrupt means and the average mental health for the same group under the hypothetical scenario that they did not participate in corruption.

### Ethics approval and consent to participate

All data used in the current study are secondary sources of data and freely available in the public domain. The ethics approval and consent to participate of this study was not applicable. All the methods were carried out in accordance with relevant guidelines and regulations.

## Results

### Descriptive statistics

Table 1 presented the descriptive statistics. We divided the samples into a treatment group and a control group, representing those who attended junior high school through corrupt means and those who did not. It is worth noting that the number of students attended junior high school through corrupt means was not negligible, the treatment group consists of 2440 adolescents, which accounts for 14.142% of the total sample. Furthermore, there were significant differences in mental health between the treatment group and the control group. Students who gained admission through corrupt means exhibited poorer mental health, and the difference was statistically significant at the 1% level.

**Table 1:** Descriptive statistics.

Variable	Treatment group		Control group		Difference	
	Mean	S.d	Mean	S.d	Diff	t-stat
Mental health	19.17	4.197	19.70	4.020	−0.527***	−5.973
Corruption	1	0	0	0	–	–
Age	14.06	1.383	13.84	1.317	0.219***	7.56
Gender	0.531	0.499	0.487	0.500	0.044***	4.015
Nationality	0.876	0.329	0.922	0.268	−0.046***	−7.538
Test scores	67.00	14.99	69.10	13.12	−2.101***	−7.178
Grade	0.516	0.500	0.464	0.499	0.051***	4.686
Family economic condition	2.842	0.627	2.811	0.584	0.031**	2.426
Parents' education level	4.140	2.043	4.134	1.992	0.006	0.135
Number of students within class	50.58	13.48	48.19	12.65	2.387***	8.554
Class rank within school	3.341	0.961	3.413	0.961	−0.072***	−3.419
School rank within district	4.016	0.844	3.953	0.821	0.064***	3.537
Observations	2440		14,814			

Covariates also revealed differences between the two groups. On average, adolescents admitted through corrupt means were older, in higher grades, had worse test scores, and were more likely to be male and ethnic minorities. Furthermore, their family economic conditions were relatively affluent, and there were more students in their class. However, although it showed that corruptly admitted students came from relatively higher-ranked schools, their class ranking within the school was relatively low.

### Matching results

In Table 2, we displayed the bias before and after matching, all the standardized bias of covariates, except for parents’ education level, has significantly reduced. On average, the standardized bias has reduced from 10.4 to 2%, that is, 80.8% of the bias has been reduced due to the matching. Rubin's B is an indicator that measures the absolute standardized difference between the means of the linear propensity score index in the treated and non-treated groups, after matching, the value of Rubin's B was 6.6. For the samples to be considered sufficiently balanced, it is recommended that Rubin's B be lower than 25^23^. In sum, matched individuals exhibited a high degree of similarity with regard to covariates, implying favorable outcomes of the matching procedure.

**Table 2:** Balancing tests.

	Bias		
	Before matching (%)	After matching (%)	Reduction (%)
Age	16.2	2.5	84.3
Gender	8.8	−1.4	84.1
Nationality	−15.2	−2.6	83
Test scores	−14.9	−1.5	90.3
Grade	10.2	1.5	85.6
Family economic condition	5.2	−3.9	25.3
Parents' education level	0.3	−4.4	−1407.6
Number of students within class	18.3	−2	89.3
Class rank within school	−7.5	−0.3	96
School rank within district	7.7	0	100
All covariates	10.4	2	80.8
Rubin's B	32.9	6.6	

Matching method: 1:1 nearest neighbor with replacement.

**Figure 1:**
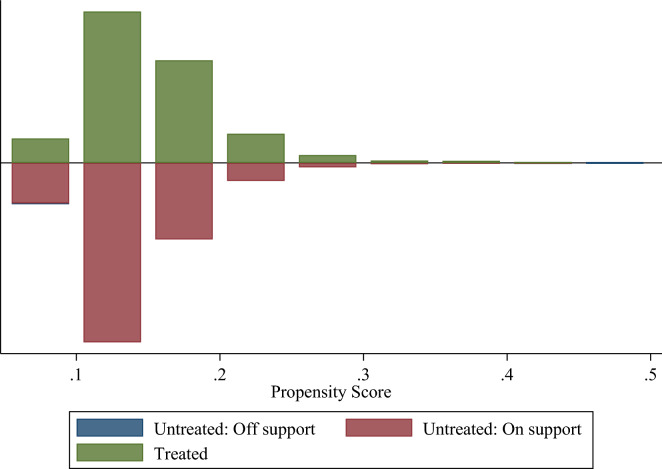
Propensity score distribution and common support. “Treated” indicates those who attended junior high school through corrupt means. “Untreated: Off support” indicates those without corrupt that do not have a suitable match. “Untreated: On support” indicates those without corrupt that have a suitable match.

The propensity score distribution and common support area were shown in Fig. 1. All 2440 treatment group samples had suitable matches, while very few individuals in the control group did not have a suitable match and would be excluded from subsequent analysis. Importantly, matched individuals in the treatment and control groups had similar propensity scores, indicating that the quality of the matching is reliable.

### Impact of corruption on adolescent mental health

The impact of corruption on adolescent mental health were shown in Table 3. Attending junior high school through corrupt means has a significant negative impact on adolescent mental health. The average treatment effect on the treated (ATT) was −0.273 and significant at the 5% level in the 1:1 nearest neighbor matching. This indicated that the mental health of those who attended school through corrupt means is worse by 1.402%. When we expanded the matching scope by using 1:4 nearest neighbor matching to match more samples in the control group, the ATT was −0.388 and significant at the 1% level, indicating that the mental health of those who attended school through corrupt means was worse by 1.984%. We also reported the results of Radius matching and Kernel matching in Table 3. The ATT values were −0.511 and −0.462, respectively, and both were significant at the 1% level. Generally, when employing various matching methods, the ATT values are consistently negative. This implies that the mental health of students admitted through corrupt practices significantly deteriorates compared to what it would be if their admission had not been through corrupt means. This demonstrates the adverse impact of corrupt admissions on mental health.

**Table 3:** Effect of corruption on adolescent mental health.

Matching methods	Mean treated	Mean control	ATT	% effect	N
1:1 nearest neighbor matching	19.169	19.442	−0.273**	−1.402	17,247
			(0.126)		
1:4 nearest neighbor matching	19.169	19.558	−0.388***	−1.984	17,247
			(0.102)		
Radius matching	19.169	19.680	−0.511***	−2.597	17,247
			(0.091)		
Kernel matching	19.169	19.632	−0.462***	−2.353	17,247
			(0.092)		

*P < 0.1, **P < 0.05, ***P < 0.01. Values in parentheses are standard errors. Mean Treated: after matching mean of treatment group. Mean Control: after matching mean of control group. ATT: mean of the treatment group—mean of the control group. N: number of observations in the common support area.

### Mechanisms

Our research has demonstrated the negative effect of corruption on adolescent mental health. However, further exploration is needed to reveal the mechanisms through which it exerted the impacts. Table 4 presents the results, we conducted mediation effect tests following the method proposed by Baron and Kenny (1986)^24^. We first regressed the independent variable on the dependent variable, then regressed the independent variable on the mediator. Finally, we regressed both the independent variable and the mediator on the dependent variable simultaneously. Using ordinary least squares regression, we reported the impact of corruption on adolescent mental health in Column (1) of Table 4, with a significantly negative coefficient (coefficient = −0.425, *p* value < 0.01). The results in Column (2) show that corruption in school admissions does not have a significant impact on adolescents' interpersonal relationships, and the Sobel test is also not significant, although the results in Column (3) indicate that adolescents' interpersonal relationships affect mental health. This suggests that corruption in school admissions does not impact mental health through adolescents' interpersonal relationships.

**Table 4:** Results of mediation test.

		Mediator: Number of friends		Mediator: Teachers' criticism received by student			Mediator: Teachers' criticism received by parents		Mediator: Desire to leave this school	
	(1)	(2)	(3)	(4)	(5)		(6)	(7)	(8)	(9)
	Mental health	Mediator	Mental health	Mediator	Mental health		Mediator	Mental health	Mediator	Mental health
Corruption	−0.425***	0.032	−0.414***	0.041***	−0.392***		0.079***	−0.373***	0.100***	−0.319***
	(0.088)	(0.319)	(0.089)	(0.015)	(0.087)		(0.018)	(0.088)	(0.019)	(0.086)
Mediator			0.013***		−0.819***			−0.591***		−1.038***
			(0.002)		(0.044)			(0.038)		(0.035)
Controls	Y	Y	Y	Y	Y		Y	Y	Y	Y
Sobel test		0.101		−2.708***			−4.286***		−5.287***	
Proportion of mediating effect		0.001		0.080			0.111		0.246	
R^2^	0.028	0.040	0.030	0.044	0.047		0.037	0.041	0.032	0.075
N	17,254	16,831	16,831	17,152	17,152		17,130	17,130	17,148	17,148

*P < 0.1, **P < 0.05, ***P < 0.01.

Teacher criticism is an important mechanism through which corruption in school admissions affects adolescents' mental health. The results in Column (4) of Table 4 show that corruption in school admissions significantly increases the likelihood of students being criticized by teachers. The results in Column (5) indicate that teacher criticism has a significant negative impact on mental health, and the coefficient for corruption in school admissions (coefficient = −0.392) is smaller than in Column (1) (coefficient = −0.425). Similarly, the results in Column (6) show that corruption in school admissions also significantly increases the likelihood of students' parents being criticized by teachers. The results in Column (7) indicate that teacher criticism of students' parents has a significant negative impact on mental health, and the coefficient for corruption in school admissions (coefficient = −0.373) is smaller than in Column (1) (coefficient = −0.425) indicating that teacher criticism is an effective mediating variable. The Sobel tests for these two mechanisms are significant at the 1% level, with the mediation effects accounting for 8% and 11.1% of the total effect, respectively.

Adolescents who were admitted to schools by corrupt means had a lower sense of identity with the school. The results in Column (8) of Table 4 show that corruption in school admissions significantly increases students' desire to leave the school. Column (9) indicates that, after including this mediator, the coefficient for corruption is lower than in Column (1). Additionally, the Sobel test is significant at the 1% level, with the mediation effect accounting for 24.6% of the total effect. Therefore, students admitted through corruption lack a sense of belonging to the school and desire to leave, which negatively impacts their mental health.

## Discussion

To our knowledge, this is the first study to explore the impact of corruption on adolescent mental health. Given the limited educational resources and the opaqueness of political systems in developing countries like China, the education sector is particularly susceptible to corruption. We have uncovered evidence that corruption in junior high school admissions can harm adolescent mental health. By using a nationally representative sample of Chinese adolescents, we revealed that those who attended junior high school through corrupt means had worsened mental health. Different matching algorithms of PSM were employed and demonstrated the robustness of the results. We employed four matching methods, including 1:1 nearest neighbor matching, 1:4 nearest neighbor matching, radius matching, and kernel matching. All these different matching methods yielded consistent results. The previous research on the topic of corruption has mainly focused on the employees in public sectors, and the development of the economy^25–27^. However, there has been relatively little attention paid to the issue of corruption during school admission in developing economies. Our study fills this research gap and provides a new perspective to understand the mental health problems among Chinese adolescents.

Corruption engenders asymmetrical outcomes, whereby certain individuals reap benefits at the expense of others. We argued that even for adolescents who benefited from corruption, it may not lead to the phenomenon of "winner takes all". Although those who attended junior high school by corrupt means may gain benefits, cognitive dissonance theory suggests that people have a natural tendency to seek consistency between their beliefs and actions, when there is a conflict between these elements, it creates a state of mental discomfort^28^. For adolescents who have benefited from corruption, their belief in fairness, which stems from basic educational concepts, inspiring the idea that unfairly enrichment through corruption is entirely wrong. However, since they gained admission by corrupt means, the conflict between their belief and behavior created negative mental health effects. We proposed an extension of the cognitive dissonance theory by providing insight into the psychological mechanisms underlying the conflict between moral values and corrupt experience among adolescents.

We have revealed some of the mechanisms by which corruption can have a negative impact on adolescent mental health. Existing literature mainly explored the mechanisms from the perspective of economic or income fluctuations^2^. Focused on adolescents, this research provides a new angle to understand why corruption can hurt their mental health. We found that corruption did not have a significant impact on interpersonal relationships. One possible reason is that classmates may not be aware of each other's admission processes, and friendships are often based on common interests and hobbies. Teacher criticism is the primary mechanism, they play a crucial role in maintaining justice and may inclined to strive to completely eradicate corrupt practices and ensure that everyone in the class is enrolled through fair and legal means. However, the current educational system is difficult to change dramatically in the short term, which means that corrupt practices during the admission process may persist for some time. Teachers themselves are unable to change this situation. As a means of upholding justice, teachers tended to offer more criticism towards those who benefited from corrupt practices as a form of psychological compensation. Moreover, criticism is a common means for teachers to provide negative feedback, it may trigger a series of negative mental health outcomes, including undermining adolescents' self-concept and sense of worth^29,30^. Chinese students are particularly sensitive to negative feedback from teachers, which may lead them to depreciate their own abilities^31^, trigger defense mechanisms, resulting in feelings of resentment^32^, and in severe cases, even mental health disorders^33^. Moreover, when teachers criticize students' parents, it may be perceived as a punishment for engaging in corrupt practices, causing increased psychological distress and evoking feelings of shame among adolescents, amplifying the negative psychological impacts generated by cognitive dissonance. Remaining in the current situation becomes a psychological torment, to ease this punishment, they are more inclined to leave the current school. Therefore, these punishments and psychological costs have exacerbated the mental health of adolescents.

This paper contributes to a broad literature focused on justice in the school environment. The first strand used physical environmental justice and found environmental risks, such as poor air quality, poor community design, or contaminated playgrounds, could lead to respiratory illness, and poor performance in school^34,35^. The second strand focused on perceived school justice, and examined its correlation with students’ engagement, academic performance, reported bias-based bullying, and school violence^36–39^. This article differs from the existing literature as we studied the negative effects of ex-ante injustice in junior high school, and we demonstrated the importance of educational justice from a new perspective.

This research echoes the significance of anti-corruption efforts in education sectors, inattention to corruption in school selection will place all other aspects of a nation’s economic and social ambitions at risk^40^. Empirical evidence has revealed that anti-corruption campaigns in China have effectively reduced the BMI and overweight rates of public sector employees^25^, while similar campaigns in Vietnam have had substantial positive impacts on the mental health of adults^2^. Our results suggest that the actions of anti-corruption in junior high school admissions might improve adolescent mental health.

Although China has a relatively sound legal system to combat corruption in admissions, corruption in the admissions field still occurs and is reported frequently. The lack of sufficient constraints and supervision over the power of education officials is the root cause of corruption, while the unequal distribution of educational resources exacerbates the likelihood of such corruption occurring. Measures to curb admission corruption should be strengthened. First, it is crucial to improve the institutional framework for combating corruption in the education sector, which can be achieved by implementing transparent and equitable admission procedures and imposing stringent penalties for corrupt activities. Second, the financial support for basic education should be distributed fairly in different schools, which can reduce the gap in school educational facilities. Third, promoting teacher rotation between schools is necessary for the equalization of teacher resources. In sum, the findings of this paper underscore the importance of developing countries prioritizing efforts, and implementing timely and effective measures to ensure equity within the education sector.

This study has limitations that should be noted. First, it should be noted that corruption in China varies significantly across different regions, and the presence of a large number of internal migrants may lead to diverse educational resource demands in different areas, highlighting the significance of exploring regional differences. However, the data used in this study does not provide comprehensive geographic information, which hinders us to examine the impact of corruption based on regional variations. Second, due to the strict confidentiality of personal information in the questionnaire, and the fact that corrupt admission behavior will not be subject to subsequent punishment after admission, parents have no need to systematically provide incorrect answers to this question. Nevertheless, it must be acknowledged that there may still be some parents who did not provide truthful answers. Third, adolescent mental health trauma can persist over an extended period^41^, and have a profound impact on an individual's development. Examining the long-term mental health effects of corruption are both interesting and meaningful. Unfortunately, the limited sample periods provided by the China Education Panel Survey (CEPS) make it impractical to uncover this phenomenon at present.

## Data Availability

The data used in this paper is from the China Education Panel Survey (CEPS). The official direct accessible link to the survey is http://www.cnsda.org/index.php?r=projects/view&id=72810330.
